# Low impact of regular PCR testing on presence at work site during the COVID-19 pandemic: experiences during an open observational study in Lower Saxony 2020-21

**DOI:** 10.1186/s12889-023-15036-9

**Published:** 2023-02-03

**Authors:** Lisa K. Seiler, Susanne Stolpe, Nils Stanislawski, Frank Stahl, Martin Witt, Rebecca Jonczyk, Stefanie Heiden, Holger Blume, Bernd Kowall, Cornelia Blume

**Affiliations:** 1 Institute of Technical Chemistry, Leibniz University Hannover, Hanover, Germany; 2 Institute for Clinical Epidemiology, University Hospital Essen, Essen, Germany; 3 Institute of Microelectronic Systems, Leibniz University Hannover, Hanover, Germany; 4 Institute of Innovation Research, Technology Management & Entrepreneurship, Leibniz University Hannover, Hanover, Germany

**Keywords:** Workplace safety, COVID-19, PCR test, Home Office

## Abstract

**Background:**

Since social distancing during the COVID-19-pandemic had a profound impact on professional life, this study investigated the effect of PCR testing on on-site work.

**Methods:**

PCR screening, antibody testing, and questionnaires offered to 4,890 working adults in Lower Saxony were accompanied by data collection on demographics, family status, comorbidities, social situation, health-related behavior, and the number of work-related contacts. Relative risks (RR) with 95 % confidence intervals were estimated for the associations between regular PCR testing and other work and health-related variables, respectively, and working on-site. Analyses were stratified by the suitability of work tasks for mobile office.

**Results:**

Between April 2020 and February 2021, 1,643 employees underwent PCR testing. Whether mobile working was possible strongly influenced the work behavior. Persons whose work was suitable for mobile office (mobile workers) had a lower probability of working on-site than persons whose work was not suitable for mobile office (RR = 0.09 (95 % CI: 0.07 – 0.12)). In mobile workers, regular PCR-testing was slightly associated with working on-site (RR = 1.19 (0.66; 2.14)). In those whose working place was unsuitable for mobile office, the corresponding RR was 0.94 (0.80; 1.09). Compared to persons without chronic diseases, chronically ill persons worked less often on-site if their workplace was suitable for mobile office (RR = 0.73 (0.40; 1.33)), but even more often if their workplace was not suitable for mobile office (RR = 1.17 (1.04; 1.33)).

**Conclusion:**

If work was suitable for mobile office, regular PCR-testing did not have a strong effect on presence at the work site.

**Trial registration:**

An ethics vote of the responsible medical association (Lower Saxony, Germany) retrospectively approved the evaluation of the collected subject data in a pseudonymized form in the context of medical studies (No. Bo/30/2020; Bo/31/2020; Bo/32/2020).

**Supplementary Information:**

The online version contains supplementary material available at 10.1186/s12889-023-15036-9.

## Background

A significant problem with preventive measures such as social distancing against the spread of a pandemic is the social and economic collateral damage caused by the strict isolation of groups of people. In Italy, strict isolation of significant parts of the population led to a considerable reduction in COVID-19-positive patients and those severely affected [1, 2] but was associated with significant economic and social consequences. As soon as more targeted information was available on how widespread positivity for the pandemic-causing pathogen (i.e., SARS-CoV-2) is in different population groups, more targeted measures were taken that led to a definition of SARS-CoV-2 risk groups [3]. In this context, it is crucial that diagnostic measures used are effective to guarantee the medical liability also for medical health professionals with respect for vaccination procedures [4].

In an early population-based study on SARS-CoV-2 prevalence in a large German municipality not affected by a super spreading event, only one in four SARS-CoV-2 infections in private households was reported and known to the health authorities [5]. Social isolation of the general population caused unsustainable social and economic conditions in the long term. Others show, for example, that specific employees, such as teachers in a similar situation as university docents with frequent social contacts, had a particular load of stress due to the necessity of social distancing during the lockdown, preventing them from their educational mission [6]. There are also large differences in management of prevention campaigns and protocols used to reduce the risk of spreading SARS-CoV-2 across different countries [7]. Remote working was also a question of gender, as has recently been shown by Bezak et al.: Females were more stressed professionally, socially, and personally than males, which was partially caused by employers´ high expectations. Working from home here was shown to be a challenge, complicated by a lack of preparedness with the rapid start of the lockdown measures [8]. Gibbs et al. documented with a study on 112 desk workers that working from home was often associated with non-work sedentary resulting in declining physical functionality and worsened sleep quality [9]. Buonsenso et al. showed that a significant percentage of previously infected subjects reported not feeling fully recovered at follow-up or a change in their job status highlighting the requirement for successful prevention campaigns and assessment of post-acute COVID-19 sequelae [10].

In this project, PCR-screening for SARS-CoV-2 infectivity was carried out in groups of populations with an increased risk for infection based on their medical risk factors or workplace (e.g., due to frequent contact with other people). Untargeted testing for SARS-CoV-2 infectivity was carried out to enable more movement freedom for individuals, especially for more presence at their workplace. Untargeted PCR testing at the time of the study start was not recommended by the Robert Koch Institute (RKI) [11] since resources for this diagnostic measure were insufficient at the beginning of the pandemic and should be reserved for symptomatic individuals with suspicion of COVID-19. PCR-testing requires expensive laboratory instrumentation and highly skilled laboratory personnel. In addition, due to logistic reasons, several days were initially needed until the results were available [5].

The study presented here comprised selected groups of participants from several occupational groups in Lower Saxony from April 2020 to February 2021. We hypothesized that for the working world, in particular, rapid and highly precise detection of infectiousness is helpful to prevent the spreading of the virus during working in presence. Without testing, we presumed that many employees would have to work from home for preventive reasons or an increased infection risk caused by frequent unavoidable professional contacts on-site. Particularly in the case of work processes that can only be carried out poorly in the “home office”, this would also be economically significant. By testing, the employer could thus continuously operate or reopen essential sectors of the company’s work. The study aimed to show that free and voluntary tests offered to employees at a workplace could help to avert economic damage due to missing workforce on-site, despite the additional costs associated with PCR-testing. Of note, there was an increase from 15 % of employees working from home pre-pandemic compared to 66 % since the pandemic’s start [12].

We primarily aimed to address whether repetitive testing of non-symptomatic subjects in the context of the SARS-CoV-2 pandemic positively affected working on-site. Moreover, we aimed to examine the impact of further factors like health status, smoking, social problems, and risk perception on presence at the work site.

## Materials and methods

### Study design

In the context of infection prevention, mobile screening facilities for offering voluntary COVID-19 PCR tests and antibody detection tests for employees were positioned close to the workplaces of participating institutions [13]. The utilized test procedures have been described before by Corman et al. and Jonczyk et al. [14, 15]. Inclusion criteria for the study were the following: employment in one of the participating companies or institutions; age ≤ 65 years; at least one COVID-19 test during the study period; verified contact data. Due to the limited testing capacity, not all members of the occupational groups could be tested. Therefore, each institution’s respective employer or head initially invited persons to participate in free testing. The initial invitation was restricted to persons with an increased risk for a SARS-CoV-2 infection (following the national health plan (RKI) recommendations such as frequent contact with other persons or comorbidities) and then extended to walk-ins. We did not interfere with this selection, and remaining capacities were offered to walk-ins of employees on-site. Participating parties were a biotechnological company located in the southern part of Lower Saxony with mostly belt workers but also a percentage of supervisors or administrative staff, the Leibniz University Hannover (LUH) with some PhD students but mostly older aged scientists, lecturers predominantly from the natural science or the (social-) economics or the engineering or the math and physics-department and administrative staff, a theatre (actors) and several nursing homes (nurses) and schools (mostly teachers, some juvenile pupils aged 16 years and older) in Hannover (Tab. S1). No feedback was given to the respective employer on whether a selected person used the offer for regular testing. Furthermore, employees could also voluntarily participate in testing if additional capacities remained. Information on gender, age, and risk factors were collected.

### Questionnaire

**Fig. 1:**
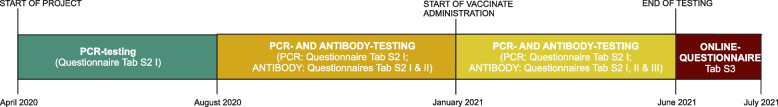
Schematic illustrating the study design with a pre-phase of PCR-only-testing (April to August 2020) with a questionnaire concerning RKI-criteria (questionnaire Tab. S2 I, see supplement) and a main-phase of PCR- and antibody testing in parallel to an additional questionnaire (questionnaire Tab. S2 II, see supplement; August 2020 to June 2021) and a post-phase after testing with questionnaire Tab. S2 III

In parallel to PCR-testing and antibody testing, for each test, participants answered a questionnaire regarding contacts with COVID-19-positive persons, symptoms of a COVID-19-infection, chronic diseases (questionnaire Tab. S2 I). Starting in August 2020, questions on subjective health, emotional stress, and health behavior were included in the questionnaire and answered by those participants receiving not only PCR, but also antibody testing. This questionnaire Tab. S2 II covered the following topics: (i) worries about health or due to problems related to family and friends or work or due to financial difficulties, change of behavior regarding health service utilization (refraining from seeing a doctor in case of feeling sick, canceling of appointments or use of telephone consulting) during the last six months and self-assessment of the risk for corona infection (0 = no risk, 5 = high risk). After the test period, all participants with known email-address received an additional online questionnaire (Tab. S3, see supplement) in August 2021. These participants were asked about workplace characteristics before the pandemic and during the testing period about suitability for mobile work (that means that the employee can do his job from any place but not necessarily on-site) and risk of infection due to frequent contact with coworkers, customers, or pupils. The schedule of the questionnaires is shown in Fig. 1.

### Variable definitions

Working primarily on-site was coded with ‘yes’ if the participants worked two days or less at home. The type of workplace was coded according to the workplace at which participants worked most of the week. Suitability of work for mobile working was coded ‘yes’ if it was rated as suitable or primarily suitable by participants. Regular testing was defined as at least three tests with a maximal time lag of 14 days each. The minimum length of a test period with regular testing was 28 days. Chronic diseases (diabetes, obesity, heart disease, chronic lung disease, chronic bowel disease) or COVID-19-symptoms were coded as present if ever mentioned during the test period. Feeling strongly impaired during the last six months was coded as present if participants responded accordingly to at least one out of seven questions relating to worries about health, difficulties with partner, family or friends, strain due to care for children, parents or other family, stress at work or school, financial problems, or missing close contacts. Social problems in the last three months were coded present if participants reported either feeling lonely, isolated, or excluded from the community. A change in health-related behavior (such as avoided, reduced, or canceled visits to a doctor or cancelled preventive or rehabilitation therapy or telephone consultation) was coded as present if ever mentioned during the test period.

We did a complete case analysis.

### Statistical analyses

We estimated the prevalence of participant and workplace characteristics stratified by the suitability of the workplace for mobile office. Fitting log-binomial regression models, we estimated univariate crude relative risks for working primarily on-site with 95 % confidence intervals (CI) for variables related to workplace conditions, regular testing, and personal characteristics. Further, we stratified these analyses by the suitability of the work for mobile work. We did not adjust for potential confounders [16] as we did not want to assess causal effects. All analyses were done using SAS9.4. Participants with no regular testing were the reference group of our exposure of interest, and participants with regular testing were the index group.

## Results

### Study participants

Altogether, 4,890 test persons in Lower Saxony were recorded concerning their infectivity (by PCR test) and contamination (by antibody detection tests) with SARS-CoV-2 during April 2020 and February 2021. *N* = 929 subjects participating in antibody testing answered questions regarding health, emotional stress, and health behavior. Of the 3,846 subjects invited to the online questionnaire, 1,643 subjects participated (response rate of 43 %); 53 % of participants in the online questionnaire were male. The mean age was 42.7 years (standard deviation (SD) 12.2) (Table 1). 34 % of the participants worked in university departments, 54 % at a biotechnological company, and 7% in nursing homes or schools. Three out of four participants (*N* = 1,224) rated their work suitable for a mobile office. 43 % (*N* = 714) estimated that 80 % - 100 % of their work could be fulfilled in a mobile office.

**Table 1:** Results of online questionnaire (*N *= 1,643): characteristics of work places not/only limited suitable for mobile office and suitable for mobile office

	Not/only limited suitable for mobile office (*N *= 419)	Suitable for mobile office (*N *= 1,224)	Total (*N* = 1,643)
Sex			
Male	203 (48.5)	660 (53.9)	863 (52.5)
Female	216 (51.5)	564 (40.1)	780 (47.5)
Age (mean, (SD))	42.6 (12.9)	42.7 (12.0)	42.7 (12.2)
<30	87 (20.8)	213 (17.4)	300 (18.3)
30-<50	177 (42.2)	594 (48.5)	771 (46.9)
50-65	155 (37.0)	417 (34.1)	572 (34.8)
Living in a single household	92 (22.0)	213 (17.4)	305 (18.6)
Self-rated risk for corona infection (0-5) (mean (SD)) (*N*=927))	2.58 (1.19)	2.31 (1.00)	2.39 (1.07)
Low (0-<2)	51 (19.8)	153 (22.8)	204 (22.0)
Medium (2-<3)	63 (24.5)	230 (34.3)	293 (31.6)
High (3-5)	143 (55.6)	287 (42.8)	430 (46.4)
Number of tests in test period (mean (SD))	8.8 (6.6)	8.5 (6.0)	8.6 (6.2)
1-2 tests	83 (19.8)	228 (18.6)	311 (18.9)
3-5	86 (20.5)	204 (16.7)	290 (17.7)
6-9	60 (14.3)	256 (20.9)	316 (19.2)
10-14	113 (27.0)	399 (32.6))	512 (31.2)
15 and more tests	77 (18.4)	136 (11.1)	213 (13.0)
Employer			
University	117 (27.9)	448 (36.6)	565 (34.4)
Nursery home/School	69 (16.5)	53 (4.3)	122 (7.4)
Sartorius	186 (44.4)	696 (56.9)	882 (53.7)
Other	47 (11.2)	27 (2.2)	74 (4.5)
Regular Tests^a^ no	322 (76.9)	1,069 (87.4)	1,391 (84.7)
Yes	97 (23.1)	154 (12.6)	251 (15.3)
Medical/social occupation (*N*=1.054)	224 (71.1)	456 (61.7)	680 (64.5)
Working mostly on site before pandemic- no	27 (6.4)	332 (27.1)	359 (21.9)
Yes	392 (93.6)	892 (72.9)	1,284 (78.1)
Working mostly on site during test period no	86 (20.5)	1,048 (85.6)	1,134 (69.0)
Yes	333 (79.5)	176 (14.4)	509 (31.0)
Type of working place (before pandemic)			
Single room	73 (17.4)	310 (25.3)	383 (23.3)
Multi-person room (2-4)	62 (14.8)	352 (28.8)	414 (25.2)
Room with 5 persons or more	60 (14.3)	471 (38.5)	531 (32.4)
Production/Laboratory	178 (42.5)	59 (4.8)	237 (14.4)
Sales/outdoor/logistic	46 (11.0)	32 (2.6)	78 (4.8)
Type of working place (during pandemic)			
Single room	96 (22.9)	519 (42.4)	615 (37.4)
Multi-person room (2-4)	56 (13.4)	263 (21.5)	319 (19.4)
Room with 5 persons or more	43 (10.3)	302 (24.7)	345 (21.0)
Production/Laboratory	185 (44.2)	64 (5.2)	249 (15.2)
Sales/outdoor/logistic	15 (3.6)	11 (0.9)	26 (1.6)
Part of work that can be fulfilled in home office			
0%-<50%	405 (96.7)	168 (13.7)	573 (34.9)
50%-<80%	7 (1.7)	349 (28.5)	356 (21.7)
80%-100%	7 (1.7)	707 (57.8)	714 (43.4)
Chronic diseases^b^ (*N*=1,618) no	323 (78.0)	970 (80.6)	1,293 (79.9)
Yes	91 (22.0)	234 (19.4)	325 (20.1)
Smoking (*N*=1,604) no	314 (76.0)	1,029 (86.4)	1,343 (83.7)
Yes	99 (24.0)	162 (13.6)	261 (16.3)
Covid symptoms in last 14 days – no	272 (64.9)	699 (57.2)	971 (59.1)
Yes	147 (35.1)	524 (42.8)	671 (40.9)
Change in behaviour in regard to health service utilization^c^ (*N*=924) - no	133 (52.2)	294 (44.0)	427 (46.2)
Yes	122 (47.8)	375 (56.0)	497 (53.8)
Feeling strongly impaired due to personal problems^d^ in last 3 months (*N*=929) – no	132 (51.4)	311 (46.3)	443 (47.7)
Yes	125 (48.6)	361 (53.7)	486 (52.3)
Social problems in last 3 months^e^ (*N*=929) - no	187 (72.8)	463 (68.9)	650 (70.0)
Yes	70 (27.2)	209 (31.1)	279 (30.0)

^a^Regular testing: at least 3 tests with a maximum lag time of 14 days

^b^Includes diabetes, adiposity, heart disease, chronic lung disease, chronic bowel disease

^c^includes avoided, reduced or cancelled visits to a doctor or cancellation of rehabilitation/preventive therapy or use of telephone consultation

^d^feeling impaired due to worries about health, difficulties with partner, family or friends, strain due to care for children, parents or other family, stress at work or school, financial problems or missing social close contacts

^e^Sometimes or often feeling lonely in a community or feeling excluded or isolated

### Test frequency

About 40 % of the participants in the online survey had a maximum of five tests; 13 % were tested 15 times or more. The mean number of tests was 8.6 (SD 6.2). Participants with workplaces unsuitable for mobile office were more often tested regularly (23 % vs. 13 %) (Table 2).

**Table 2:** Working mostly on site – by demographic characteristics, job characteristics, comorbidities and other factors (N, % and relative risk with 95 % confidence interval). *N* = 1,643

	working mostly on site (max. 2 days/month at home)			Crude relative risk (95 %-Confidence interval)
	Yes	No	% yes	
Gender Male	185	678	21.4	Ref.
Female	197	583	25.3	1.18 (0.99; 1.40)
Age (10 yrs)				1.02 (1.00; 1.04)
<30	66	234	22.0	Ref.
30-<50	161	610	20.9	0.95 (0.74; 1.22)
50-65	155	417	27.1	1.23 (0.96; 1.58)
Part of work that can be done effectively at home (every 10% increase)				0.64 (0.62; 0.68)
<50%	317	256	55.3	Ref.
50-70%	32	324	9.0	0.16 (0.12; 0.23)
80-100%	33	681	4.6	0.08 (0.06; 0.12)
Work suited for mobile work No	300	119	71.6	Ref.
Yes	82	1,142	6.7	0.09 (0.07; 0.12)
Employer				
Nursey home/school	55	67	45.1	Ref.
University	100	465	17.7	0.39 (0.30; 0.51)
Sartorius	191	691	21.7	0.48 (0.38; 0.61)
Other	36	38	48.7	1.08 (0.80; 1.46)
Working place during test period				
Single room	83	532	13.5	Ref.
Multiple persons room	108	556	16.3	1.21 (0.93; 1.57)
Laboratory/production	158	91	63.5	4.70 (3.77; 5.87)
Sales / field service	33	82	28.7	2.13 (1.50; 3.02)
Number of job related contacts >15min before testing				
None	12	20	37.5	Ref.
1-5 persons	119	508	19.0	0.51 (0.31; 0.81)
6 or more	251	733	25.5	0.68 (0.43; 1.08)
Regular tests^a^ no	304	1,087	21.9	Ref.
Yes	78	173	31.1	1.42 (1.15; 1.75)
Smoker No	277	1,066	20.6	Ref.
Yes	96	165	36.8	1.78 (1.47; 2.16)
Medical or social occupation no	93	281	24.9	Ref.
Yes	179	501	26.3	1.06 (0.85; 1.31)
Chronic disease No	292	1,001	22.6	Ref.
Yes	86	239	26.5	1.17 (0.95; 1.44)
Diabetes No	361	1,205	23.1	Ref.
Yes	15	29	34.1	1.48 (0.97; 2.25)
Obesity No	350	1,191	22.7	Ref.
Yes	26	44	37.1	1.64 (1.19; 2.25)
Chronic lung disease No	348	1,137	23.4	Ref.
Yes	30	102	22.7	0.97 (0.70; 1.35)
Ever Contact with Covid Case No	340	1113	23.4	Ref
Yes	41	145	22.0	0.94 (0.71; 1.25)
Covid symptoms in last 14 days No	251	720	25.9	Ref
Yes	131	540	19.5	0.76 (0.63; 0.91)
Self-rated risk of corona infection (0-5) increase by^c^ (*N*=927)				1.12 (1.01; 1.25)
Low Self-rated risk (0-<2)	48	156	23.5	Ref
Medium self-rated risk (2-<3)	62	231	21.2	0.90 (0.65; 1.25)
Higher self-rated risk (3+)	115	315	26.7	1.14 (0.85; 1.52)
Change in behaviour in regard to health service utilization^d^ (*N*=924)				
No	120	307	28.1	Ref
Yes	105	392	21.1	0.75 (0.60; 0.94)
Feeling strongly impaired due to personal problems^e^ in last 3 months (*N*=929)				
No	112	331	25.3	Ref
Yes	114	372	23.5	0.93 (0.74; 1.16)
Social problems^f^ in last 3 months (*N*=929)				
No	63	163	31.7	Ref
Yes	136	567	22.3	0.71 (0.55; 0.90)

^a^Regular testing: at least 3 tests with a maximum lag time of 14 days

^b^Includes diabetes, obesity, heart disease, chronic lung disease, chronic bowel disease

^c^A rating of 0 indicates no self-assessed risk, a rating of 5 indicates a very high self-assessed risk of corona infection

^d^Includes avoided, reduced or cancelled visits to a doctor or cancellation of rehabilitation/preventive therapy or use of telephone consultation

^e^feeling impaired due to worries about health, difficulties with partner, family or friends, strain due to care for children, parents or other family, stress at work or school, financial problems or missing social close contacts

^f^Sometimes/often feeling lonely in a community or feeling excluded or feeling isolated

### Testing and working on-site

Working on-site was reduced during the pandemic compared to pre-pandemic times, from 94 % to 80 % in the group with work unsuitable for mobile office and from 73 % to 14 % in those with suitable work (Table 1). Suitability of the work for mobile office was most strongly associated with presence at work (RR = 0.09 (0.07; 0.12)) (Table 2). In mobile workers, regular PCR-testing was slightly associated with working on-site (RR = 1.19 (0.66; 2.14)). In those whose workplace was not suitable for mobile office, the corresponding RR was 0.94 (0.80; 1.09) (Table 3).

**Table 3:** Working mostly on site – by demographic characteristics, job characteristics, comorbidities and other factors (N, % and relative risk with 95 % confidence interval) - stratified by suitability for mobile work. *N* = 1,643

	Job suitable for mobile office (*N*=1,224)Working mostly on site		Job not suitable for mobile office (*N*=419)Working mostly on site	
	N (%)	RR (95%-CI)	N (%)	RR (95%-CI)
Gender				
Male	32 (4.9)	Ref.	153(75.4)	Ref.
Female	50 (8.9)	1.83 (1.19; 2.81)	147 (68.1)	0.90 (0.80; 1.02)
Age (years)				
<30	15 (7.0)	Ref.	51 (58.6)	Ref.
30-<50	31 (5.2)	0.74 (0.41; 1.35)	130 (73.5)	1.25 (1.03; 1.53)
50-65	36 (8.6)	1.23 (0.69; 2.19)	119 (76.8)	1.31 (1.08; 1.59)
Part of work that can be done effectively at home (every 10% increase)	-	0.77 (0.71; 0.84)	-	0.73 (0.67; 0.80)
<50%	26 (15.5)	Ref.	291 (71.9)	Ref.
50-70%	29 (8.3)	0.54 (0.33; 0.88)	3 (42.9)	0.60 (0.25; 1.41)
80-100%	27 (23.8)	2.18 (1.21; 3.62)	6 (85.7)	1.19 (0.88; 1.62)
Employer				
Nursey home/school	12 (22.6)	Ref.	43 (62.3)	Ref.
University	34 (7.6)	0.34 (0.19; 0.61)	66 (56.4)	0-91 (0.71; 1.15)
Sartorius	31 (4.5)	0.20 (0.11; 0.57)	160 (86.0)	1.38 (1.14; 1.67)
Other	5 (18.5)	0.82 (0.32; 2.08)	16 (66.0)	1.06 (0.80; 1.39)
Number of job related contacts >15min duration - before testing period				
None	2 (10.0)	Ref.	10 (83.3)	Ref.
1-5 persons	34 (6.7)	0.67 (0.17; 2.59)	85 (71.4)	0.86 (0.65; 1.13)
6 or more	46 (6.6)	(0.17;2.53)	205 (17.2)	0.85 (0.66; 1.11)
Regular tested^a^ No	70 (6.6)	Ref.	234 (72.7)	Ref.
Yes	12 (7.8)	1.19 (0.66; 2.14)	66 (68.0)	0.94 (0.80; 1.09)
Living alone No	62 (6.1)	Ref.	235 (71.9)	Ref.
Yes	20 (9.4)	1.53 (0.94; 2.47)	65 (70.7)	0.98 (0.85; 1.14)
Current Smoker No	62 (6.0)	Ref.	80 (80.8)	Ref.
Yes	16 (9.9)	1.64 (0.97; 2.77)	215 (68.5)	1.18 (1.04; 1.33)
Medical or social occupation no	20 (7.1)	Ref.	73 (80.2)	Ref.
Yes	41 (9.0)	1.27 (0.76; 2.13)	138 (61.6)	0.77 (0.66; 0.89)
Chronic disease^b^ No	68 (7.0)	Ref.	224 (69.4)	Ref.
Yes	12 (5.1)	0.73 (0.40; 1.33)	74 (81.3)	1.17 (1.04; 1.33)
Diabetes No	77 (6.6)	Ref.	284 (71.5)	Ref.
Yes	2 (7.1)	1.08 (0.28; 4.19)	13 (81.3)	1.14 (0.89; 1.44)
Obesity No	76 (6.6)	Ref.	274 (70.3)	Ref.
Yes	3 (6.4)	0.97 (0.32; 2.95)	23 (100)	1.42 (1.33; 1.52)
Chronic lung disease No	75 (6.8)	Ref.	273 (71.5)	Ref.
Yes	5 (5.0)	0.74 (0.30; 1.78)	25 (78.1)	1.09 (0.90; 1.33)
Self-rated risk of corona infection^c^				
0-<2	11 (7.2)	Ref.	37 (72.6)	Ref.
3-	19 (8.3)	1.15 (0.56;2.35)	43 (68.3)	0.94 (0.74; 1.19)
4-5	27 (9.4)	1.31 (0.67; 2.57)	88 (61.5)	0.84 (0.69; 1,05)
Contact with Corona Case no	73 (6.7)	Ref.	267 (72.8)	Ref.
Yes	8 (6.0)	0.88 (0.44; 1.80)	33 (63.5)	0.87 (0.70; 1.08)
Change in behaviour in regard to health service utilization^d^ (*N*=924) no	25 (8.5)	Ref.	95 (56.9)	Ref.
Yes	33 (8.8)	1.03 (0.63; 1.70)	72 (43.1)	0.83 (0.69; 0.99)
Social problems^e^ in last 3 months(*N*=929)No	17 (12.1)	Ref.	46 (79.3)	Ref.
Yes	41 (7.7)	0.64 (0.38; 1.09)	122 (61.3)	0.77 (0.65; 0.92)

^a^Regular testing: at least 3 tests with a maximum lag time of 14 days

^b^Includes diabetes, adiposity, heart disease, chronic lung disease, chronic bowel disease

^c^A rating of 0 indicates no self-assessed risk, a rating of 5 indicates a very high self-assessed risk of corona infection

^d^Includes avoided, reduced or cancelled visits to a doctor or cancellation of rehabilitation/preventive therapy or use of telephone consultation

^e^Sometimes or often feeling lonely in a community or feeling excluded or feeling isolated

14 % of participants whose jobs were not or less suitable for mobile office did not work on-site any more during the pandemic. Compared to those participants with jobs less suitable for mobile office who worked on-site during the pandemic, these participants working at home were more often male (50 % vs. 34 % in the group working on-site). Nearly half of them were employed in industry (47 %), while among those without a job suitable for mobile office, but working at home during the pandemic, 54 % were from university. Persons working at home without a job suitable for it were older (19 % aged less than 30 years vs. 33 %), more often working in production or laboratory (45 % vs. 34 %). Regarding their morbidity, persons working in mobile office in jobs not suitable for it, more often had a heart disease (6 % vs. 2 %) or obesity (5 % vs. 0 %). However, they less often rated their danger for a corona-infection as high (52 % vs. 63 %).

### Demographics and working on-site

Women were more often present at their workplace than men (RR = 1.18 (0.99; 1.40)), and participants aged 50–65 years were more often present at their workplace than younger persons (RR = 1.23 (0.96; 1.58)) (Table 2). Generally, associations between participants` and work characteristics, respectively, and working onsite often differed in strata of work suitability for mobile office.

### Family status, lifestyle factors, comorbidity, and working on-site

Current smokers were more likely to be present on-site regardless of the suitability of their workplace for a mobile office (RR = 1.64 (0.97; 2.77) for jobs suitable for mobile office, RR = 1.18 (1.04; 1.33) for jobs not suitable for mobile office) (Table 3). In participants with jobs unsuitable for mobile office, the prevalence of chronic conditions was associated with higher workplace presence (RR = 1.17 (1.04; 1.33)) than in participants with a workplace suitable for a mobile office (RR = 0.73 (0.40; 1.33)) (Table 3). In addition, participants living alone were more often present on-site even if their job was suitable for mobile office than those who reported not living alone (RR = 1.53 (0.94; 2.47)) (Table 3).

### Missing values

Some questions we analyzed were only asked during an antibody test. As not all participants underwent antibody testing, the questions on subjective health, emotional stressors, and health behavior had more missings (*N* = 714).

### Risk assessment and working on-site

In persons with jobs suitable for mobile office, presence at work was more frequent the higher the self-rated risk of corona-infection was (RR = 1.15 (0.56; 2.35) for medium self-rated risk, and RR = 1.31 (0.67; 2.57) for high self-rated risk compared to low self-rated risk) (Table 3). Furthermore, among participants with a job unsuitable for mobile office, persons who reported a change in their health-related behavior caused by the pandemic were more seldom on-site (RR = 0.87 (0.70; 1.08)).

## Discussion

The strongest predictor for working on-site was the lack of suitability of the workplace for mobile office. Regular PCR-testing did not substantially impact on presence at the work site. In those with jobs suitable for mobile office, regular tests only led to a slight increase in work site presence.

Mobile office was more frequent in subjects that had reported that their work is generally associated with more frequent contacts with others for a period lasting longer than 15 min at work. In addition, absence from work was associated with altered health-related behaviors, clustered social problems, and experienced contact with COVID-19 cases. The above factors were further related to actual or perceived stress from the pandemic. Participants who feel more stressed seem to prefer to work at home, regardless of whether their workplace allows them to perform their job from home.

People with lower social status may not be able to set up a workplace at home because they do not have enough space or the financial resources to do so. They may also be less likely to have the opportunity in their work context to decide for themselves whether to work at home. Current smoking was associated with a higher prevalence at work in all strata. One reason for this could be a relatively lower social status of smokers compared to nonsmokers [17]. However, smokers may also be more interested in social contacts. Interestingly, having an existing chronic disease was not associated with a lower prevalence at work. Because chronic diseases are more prevalent among people with lower social status [18], this factor may be the leading cause of the association. For those living alone, the higher likelihood of working locally may reflect a need for social contact otherwise limited by the official nationwide lockdown regulation.

In this work, a higher self-assessed risk of infection correlates with a higher prevalence of on-site work, with the on-site presence most likely being the cause of the self-assessed increased risk. It is worth noting that this relationship was only present among those with the option of mobile working and not among those who did not have the option of moving their workplace home. Social stress and changes in health-related behaviors due to the pandemic were associated with lower prevalence at work. People experiencing these stresses would want to avoid additional potential viral exposure at work.

### Limitations

Tested participants in the online survey do not represent the entire target group of this study. In some workplaces, such as the university, participants took care of the registration to participate in the voluntary test offer themselves. However, in some workplaces, such as the participating company and nursing homes, the respective supervisor or manager gave a recommendation to some individuals to be tested in addition to independent voluntary participation. Specific encouragement by employers to use the test may have harmed motivation to be tested over a more extended period. Only about 50 % of all tested participants took up to five tests. We have no information on the educational background of those tested, although it can be assumed that some of the associations presented reflect a person’s underlying educational background [17]. This is particularly true for the association between current smoking or obesity and on-site attendance.

Since the online questionnaire could not be delivered to all tested individuals because some had no valid email-address, the results presented here cover only a subgroup of all participants in the PCR- and antibody testing. In general, we did not use any techniques to replace missing values. A comparison between the tested population and the population that answered the online questionnaire showed that university employees were overrepresented in the online survey. We observed a lower response rate with regard to this online questionnaire in people working in nursing homes or schools. This may be explicable by the fact, that at the time of questionnaires, people might have grown weary of participating in these questionings. Many test persons had hoped that testing would give them more freedom in their daily life or would help to support a near ending for pandemic measures such as the lockdown, which was not the case.

In future pandemic situations, testing should be mandatory, especially for people who cannot work on a mobile basis with a great risk for infection, as shown for German health workers during April 2020 and April 2021 [19]. This was later practiced to some extent during the pandemic in Germany - mainly based on the SARS-CoV-2 rapid tests - albeit with comparatively lower test reliability than PCR testing [20]. Even PCR-testing is not a universal remedy due to a relatively moderate sensitivity at best, as shown by a study on inpatients in Finland [21]. Identification of vulnerable working groups with a specific need for frequent testing thus represents an essential contribution to occupational safety and health services necessary for the future [22]. It may be additionally meaningful in older populations and populations at risk, which were threatened by prolonged grief disorders upon long-lasting social isolation [23]. Consequent PCR-testing against virus spreading was proven successful by Luxembourg´s mass screening program [24] and is urgently postulated by others [25].

## Conclusion

Programs aimed at increasing workplace prevalence in situations such as the COVID-19 pandemic were primarily intended to cover workplaces with little discretion about allowing workers to work at home. Offering voluntary testing to the workforce did not affect workplace attendance among individuals whose jobs were not conducive to mobile work. Nevertheless, for participants whose jobs are suited for mobile office, regular testing lead to a slight increase in working on-site, whereas for those whose jobs were not suitable for mobile office, regular testing barely had any effect. Because the tests were used more frequently by individuals in such a workplace, they could help increase workplace safety, particularly at production sites with an exceptionally high risk of transmission through the goods produced or in service sectors with particularly vulnerable customers, such as the health sector. Prioritization of such work areas recommended by public bodies would be desirable. It may be mentioned that the company supported by PCR-testing in this project was able to expand its production unhindered by labor shortages throughout the pandemic [26]. In contrast, publications from the Netherlands and Morocco document a lack of productivity and dissatisfaction during the lockdown in working society [27, 28].

Based on the results of this study with voluntary participants, we cannot recommend whether the costs required for mass prophylactic testing justify the effort to keep certain occupational groups in attendance during future pandemics. We cannot decide if testing projects would thus replace the lockdown imposed in the SARS-CoV-2 crisis as a preventive measure against the pandemic spread in the workforce. In agreement with other groups, we value the effectiveness of testing as a means to combat pandemic scenarios. To be more effective as a preventive measure, PCR-testing probably would have had to be controlled more strictly.

## Supplementary Information

**Additional file 1:** **Table S1.** Test persons: The table shows an overview of the registered study participants that accepted the test offer. **Table S2.** Questionnaire for study participants divided in three parts (I, II, and II). **Table S3.** Online questionnaire for study participants divided in five parts (dark grey).

## Data Availability

De-identified subject data sets will be available upon written request to the corresponding author following publication.

## Abbreviations

CI: confidence interval
Fig: figure
LUH: Leibniz University Hannover
MHH: Hannover Medical School
PCR: polymerase chain reaction
RR: relative risk
RKI: Robert Koch Institute
SD: standard deviation
Tab: table
