# Supplementary material for: Low impact of regular PCR testing on presence at work site during the COVID-19 pandemic: experiences during an open observational study in Lower Saxony 2020-21
Source: BMC Public Health. 2023 Feb 3;23:240. doi: 10.1186/s12889-023-15036-9 (PMC9897879; doi:10.1186/s12889-023-15036-9)
Supplement: Supplementary file 1 — Additional file 1: Table S1. Test persons: The table shows an overview of the registered study participants that accepted the test offer. Table S2. Questionnaire for study participants divided in three parts (I, II, and II). Table S3. Online questionnaire for study participants divided in five parts (dark grey). [file 12889_2023_15036_MOESM1_ESM.docx]

**Supplement**

Table S1: Test persons: The table shows an overview of the registered study participants that accepted the test offer.

| **Subject Affiliation** | **Number of Subjects** |
| --- | --- |
| Culture and Education | 1,396 |
| Schools and Nursing Homes | 718 |
| Company | 2,776 |
| Total | 4,890 |

Table S2: Questionnaire for study participants divided in three parts (I, II and II)

| I | | |
| --- | --- | --- |
| 1 | First name | String |
| 2 | Last name | String |
| 3 | Date of birth | Date (dd.mm.yyyy) |
| 4 | e-mail address | e-mail address |
| 5 | Phone number | Phone number |
| 6 | Primary residence street/number | String |
| 7 | Primary residence postcode | Postcode |
| 8 | Primary residence city | String |
| 9 | Residence street/number | String |
| 10 | Residence postcode | Postcode |
| 11 | Residence city | String |
| 12 | Family doctor/ medical officer | String |
| 13 | Address of Family doctor/ medical officer | String |
| 14 | Health insurance | String |
| 15 | For which partner institution are you working? | String |
| 16 | Gender | Male/female/divers |
| 17 | Are you living alone? | Yes/no |
| 18 | Do you privately care for or support others who are elderly or chronically ill? | Yes/no |
| 19 | Are you working in one of the following areas/facilities? | Infotext |
| 20 | Medical area | String |
| 21 | Community facility (school, kindergarten, nursing home, university) | String |
| 22 | We would like to ask you some questions about possible infection risks and symptoms of COVID-19. | Infotext |
| 23 | Have you had contact to a confirmed SARS-CoV-2 case? | Yes/no/unknown |
| 24 | Have you had one or more of the following symptoms within the last 14 days? If yes, state in each case for how many days. | Infotext |
| 25 | Fever | Number |
| 26 | Ague | Number |
| 27 | Increased fatigue or a significantly lower ability to work under pressure | Number |
| 28 | Melalgia | Number |
| 29 | Headache | Number |
| 30 | Sore throat | Number |
| 31 | Loss of taste and smell | Number |
| 32 | Have you even been diagnosed with any of the following chronic disease by a physician? | Infotext |
| 33 | Chronic lung disease | Yes/no/unknown |
| 34 | Diabetes | Yes/no/unknown |
| 35 | Heart disease | Yes/no/unknown |
| 36 | adiposity | Yes/no/unknown |
| 37 | Chronic disease of bowels | Yes/no/unknown |
| 38 | For the following questions, it is important whether you suffer from a chronic disease such as chronic bronchitis, allergy, chronic bowel disease. Compare your current complaints with previous problems. Have you had in the last two weeks: | Infotext |
| 39 | Persistent cough | Yes/no |
| 40 | Persistent cold | Yes/no |
| 41 | Diarrhoea | Yes/no |
| 42 | Have you been out of breath faster than usual in the last two weeks (see below for explanation)? | Yes/no |
| 43 | Select yes if you:  *Become short of breath or have difficulty breathing more quickly than usual during light exertion such as walking or climbing stairs  *Experience the sensation of shortness of breath/breathlessness or shortness of breath when sitting or lying down  *Have the sensation of shortness of breath/breathlessness when getting up from bed or a chair | Infotext |
| 44 | Are you pregnant? | Yes/no/unknown |
| 45 | Do you smoke? | Yes/no |
| 46 | Are you currently taking cortisone (in tablets)? | Yes/no/unknown |
| 47 | Are you currently taking immunosuppressants? (You are taking or receiving immunosuppressants after an organ transplant, during therapy for an autoimmune disease or as part of a chemotherapy) | Yes/no/unknown |
| 48 | Have you had a flue vaccination between October 2019 and today? | Yes/no/unknown |
|  | | |
| II | | |
| 49 | Have you experienced symptoms that you might now interpret as typical of SARS-CoV-2 infection since the presumed onset of the Corona crisis in Germany (since approximately December 2019 or January 2020)? | Yes/no/unknown |
| 50 | Which symptoms exactly? | Infotext |
| 51 | Fever and feeling sick | Yes/no |
| 52 | Cough, cold | Yes/no |
| 53 | Severe fatigue and decrease in performance | Yes/no |
| 54 | Diarrhoea | Yes/no |
| 55 | Sore throat | Yes/no |
| 56 | Headache | Yes/no |
| 57 | Pain in the limbs | Yes/no |
| 58 | Test or smell disorder | Yes/no |
| 59 | Shortness of breath | Yes/no |
| 60 |  | Infotext |
| 61 | Has anyone living in the same household had similar symptoms since February 2020? | Yes/no/living alone |
| 62 | Have you knowingly had closer contact with anyone affected by COVID-19 such as that infection is likely, since December 2019/january2020? | Yes/no |
| 63 | How would you rate your own risk getting SARS-CoV-2? (0=no risk / 5=very high risk) | Number |
| 64 | Has there been a change in your behaviour regarding health issues in the last 6 months as a result of the Corona Crisis? | Infotext |
| 65 | Yes, namely: | Infotext |
| 66 | I have cancelled doctor appointment(s) for routine check-ups. | Yes/no |
| 67 | I did not go to the doctor for a minor health problem like I usually do. | Yes/no |
| 68 | I did not go to the doctor for a more serious health problem. | Yes/no |
| 69 | My dentist/family doctor/specialist cancelled a routine appointment because of Corona. | Yes/no |
| 70 | I was unable to keep appointments for physical therapy/occupational therapy/logopaedics/or similar. | Yes/no |
| 71 | I took advantage of a phone consultation with a doctor. | Yes/no |
| 72 | No, | Infotext |
| 73 | I have not had health problems within the last 6 months and have NOT changed my behaviour. | Yes/no |
| 74 | In the last 3 months, how often did the following statements apply to you? ([almost] Never/sometimes/often) | Infotext |
| 75 | I lack companionship. | Never/sometimes/often |
| 76 | I feel excluded. | Never/sometimes/often |
| 77 | I feel isolated from others. | Never/sometimes/often |
| 78 | In the last 3 months, how much did you feel affected by the following problem? (not/little/strong) | Infotext |
| 79 | Worry about your health. | not/little/strong |
| 80 | Difficulties with spouse/life partner/boyfriend/girlfriend. | not/little/strong |
| 81 | Burden of caring for children, parents, or other family members. | not/little/strong |
| 82 | Stress at work or school. | not/little/strong |
| 83 | Financial problems or worries. | not/little/strong |
| 84 | Having no one to discuss problems with. | not/little/strong |
| 85 | Something bad that happened recently. | not/little/strong |
|  | | |
| III | | |
| 86 | Have you ever been tested positive for SARS-CoV-2 infection in the past? | Yes/no |
| 87 | If yes: When was the test performed? | Date (dd.mm.yyyy) |
| 88 | Have you had the following symptoms as part of your infection? (0=not at all, 5=very strong) | Infotext |
| 89 | Fever and feeling sick | Number |
| 90 | Cough, sore throat | Number |
| 91 | Cold | Number |
| 92 | Severe fatigue and decrease in performance | Number |
| 93 | Diarrhea | Number |
| 94 | Headache | Number |
| 95 | Pain in the limps or joints | Number |
| 96 | Taste or smell disorders | Number |
| 97 | Breathlessness and shortness of breath | Number |
| 98 | Skin rash | Number |
| 99 | Lung inflammation | Number |
| 100 | Have you been hospitalized as part of your infection? | Yes/no |
| 101 | If yes: Were you treated in an intensive care unit? | Yes/no |
| 102 | If yes: did you need to be ventilated? | Yes/no |
| 103 | Have you had complaints following your infection? (0=not at all, 5=very strong) | Infotext |
| 104 | Recurrent cough | Number |
| 105 | Chronic cough | Number |
| 106 | Fatigue | Number |
| 107 | Physical performance reduction | Number |
| 108 | Mental performance reduction | Number |
| 109 | Joint complaints | Number |
| 110 | Taste or smell disorders | Number |
| 111 | Breathlessness and shortness of breath | Number |
| 112 | How long did the symptom phase last after your acute infection phase? | Number |
| 113 | In the meantime, have you been vaccinated against SARS-CoV-2? | Yes/no |
| 114 | If yes: when have you been vaccinated (first time)? | Date (dd.mm.yyyy) |
| 115 | If yes: Have you been vaccinated with a viral vaccine or mRNA agent? | mRNA/Vector/Unknown |
| 116 | If yes: please tell us the name of vaccine (e.g., Biontech, Moderna, Pfizer) | String |
| 117 | Thanks for your answers! | Infotext |

Table S3: Online questionnaire for study participants divided in five parts (dark grey).

| **Questions regarding your workplace** | | | | | | | | | | | |  | | | | | | | | | | |
| --- | --- | --- | --- | --- | --- | --- | --- | --- | --- | --- | --- | --- | --- | --- | --- | --- | --- | --- | --- | --- | --- | --- |
|  | | | | | | | | | | | |  | | | | | | | | | | |
| Which department are you working at? | | | | | | | | | | | |  | | | | | | | | | | |
|  | | |  | | | | | | | | |  | | | | | | | | | | |
|  | | |  | | | | | | | | |  | | | | | | | | | | |
| Please indicate the nature of your primary employment. | | | | | | | | | | | |  | | | | | | | | | | |
|  | 🞎 | Full-time employment | | | | | | | | | | | | | | | | | | | | |
|  | 🞎 | Partial retirement | | | | | | | | | | | | | | | | | | | | |
|  | 🞎 | Fixed-term employment | | | | | | | | | | | | | | | | | | | | |
|  | 🞎 | Freelancer | | | | | | | | | | | | | | | | | | | | |
|  | 🞎 | Marginal employment | | | | | | | | | | | | | | | | | | | | |
|  | 🞎 | Study (full time) | | | | | | | | | | | | | | | | | | | | |
|  | 🞎 | Apprenticeship or dual curriculum | | | | | | | | | | | | | | | | | | | | |
|  | 🞎 | Internship | | | | | | | | | | | | | | | | | | | | |
|  | 🞎 | Others (please provide more information as comment) | | | | | | | | | | | | | | | | | | | | |
|  | | | | | | | | | | | | | | | | | | | | | | |
|  | | | | | | | | | | | | | | | | | | | | | | |
| **Questions regarding home office** | | | | | | | | | | | |  | | | | | | | | | | |
|  | | |  | | | | | | | | |  | | | | | | | | | | |
| Is your job home-office ready? | | | | | | | | | | | | | | | | | | | | | | |
|  | 🞎 | No | | | | | | | | | | | | | | | | | | | | |
|  | 🞎 | Yes, with limitation | | | | | | | | | | | | | | | | | | | | |
|  | 🞎 | Yes | | | | | | | | | | | | | | | | | | | | |
|  |  |  | | | | | | | | | | | | | | | | | | | | |
| To what extent can you perform your job if you are… (1 = not at all, 10 = completely) | | | | | | | | | | | | | | | | | | | | | | |
|  | | | 1 | 2 | | 3 | | | | 4 | | | 5 | | 6 | | 7 | 8 | | 9 | | 10 |
| …in your home office? | | | 🞎 | 🞎 | | 🞎 | | | | 🞎 | | | 🞎 | | 🞎 | | 🞎 | 🞎 | | 🞎 | | 🞎 |
| …at your workplace? | | | 🞎 | 🞎 | | 🞎 | | | | 🞎 | | | 🞎 | | 🞎 | | 🞎 | 🞎 | | 🞎 | | 🞎 |
|  | | | | | | | | | | | | | | | | | | | | | | |
|  | | | | | | | | | | | | | | | | | | | | | | |
| **Questions regarding professional contact before the pandemic**  **Think back to the work situation before the pandemic (until the end of February 2020).** | | | | | | | | | | | | | | | | | | | | | | |
|  | | | | | | | | | | | | | | | | | | | | | | |
| Please indicate all types of workstations and the respective hours you have spent working there to perform your work before the end of February 2020. | | | | | | | | | | | | | | | | | | | | | | |
|  | | | | | | | | | | | | | | | | | | | | | | |
|  | | | | | not at all | | less than 4 hours | | | | 4 to 12 hours | | | 12 to 20 hours | | 20 to 28 hours | | | 28 to 36 hours | | more than 36 hours | |
| Single office | | | | | 🞎 | | 🞎 | | | | 🞎 | | | 🞎 | | 🞎 | | | 🞎 | | 🞎 | |
| Multiperson office (2-4 persons) | | | | | 🞎 | | 🞎 | | | | 🞎 | | | 🞎 | | 🞎 | | | 🞎 | | 🞎 | |
| Multiperson office (5-10 persons) | | | | | 🞎 | | 🞎 | | | | 🞎 | | | 🞎 | | 🞎 | | | 🞎 | | 🞎 | |
| Multiperson office (more than 10 persons) | | | | | 🞎 | | 🞎 | | | | 🞎 | | | 🞎 | | 🞎 | | | 🞎 | | 🞎 | |
| Production workplace  (e.g. factory hall, workshop, assembly line) | | | | | 🞎 | | 🞎 | | | | 🞎 | | | 🞎 | | 🞎 | | | 🞎 | | 🞎 | |
| laboratory workplace | | | | | 🞎 | | 🞎 | | | | 🞎 | | | 🞎 | | 🞎 | | | 🞎 | | 🞎 | |
| Outdoor / open air workplace | | | | | 🞎 | | 🞎 | | | | 🞎 | | | 🞎 | | 🞎 | | | 🞎 | | 🞎 | |
| Logistics | | | | | 🞎 | | 🞎 | | | | 🞎 | | | 🞎 | | 🞎 | | | 🞎 | | 🞎 | |
| Sales force | | | | | 🞎 | | 🞎 | | | | 🞎 | | | 🞎 | | 🞎 | | | 🞎 | | 🞎 | |
|  | | | | |  | | | | | | | | | | | | | | | | | |
| In the period until the end of February 2020: In average, how many days per month did you work in your home office? | | | | | | | | | | | | | | | | | | | | | | |
|  |  |  | | | | | | | | | | | | | | | | | | | | |
|  |  |  | | | | | | | | | | | | | | | | | | | | |
| On a normal working day, how many persons during work-hours did you get in contact with before the end of February 2020? | | | | | | | | | | | | | | | | | | | | | | |
|  | 🞎 | None | | | | | | | | | | | | | | | | | | | | |
|  | 🞎 | 1-5 persons | | | | | | | | | | | | | | | | | | | | |
|  | 🞎 | 6-10 persons | | | | | | | | | | | | | | | | | | | | |
|  | 🞎 | 11-20 persons | | | | | | | | | | | | | | | | | | | | |
|  | 🞎 | More than 20 persons | | | | | | | | | | | | | | | | | | | | |
|  |  |  | | | | | | | | | | | | | | | | | | | | |
| On a normal workday before the end of February 2020, how many people did you have contact with for more than 15 minutes? | | | | | | | | | | | | | | | | | | | | | | |
|  | 🞎 | None | | | | | | | | | | | | | | | | | | | | |
|  | 🞎 | 1-2 persons | | | | | | | | | | | | | | | | | | | | |
|  | 🞎 | 3-5 persons | | | | | | | | | | | | | | | | | | | | |
|  | 🞎 | 6-10 persons | | | | | | | | | | | | | | | | | | | | |
|  | 🞎 | More than 10 persons | | | | | | | | | | | | | | | | | | | | |
|  |  |  | | | | | | | | | | | | | | | | | | | | |
| On a normal working day before the end of February 2020, if you had contacts that lasted more than 15 minutes, in how many of these contacts could you maintain a distance of at least 1.50 m? | | | | | | | | | | | | | | | | | | | | | | |
|  | 🞎 | None | | | | | | | | | | | | | | | | | | | | |
|  | 🞎 | 1-2 persons | | | | | | | | | | | | | | | | | | | | |
|  | 🞎 | 3-5 persons | | | | | | | | | | | | | | | | | | | | |
|  | 🞎 | 6-10 persons | | | | | | | | | | | | | | | | | | | | |
|  | 🞎 | More than 10 persons | | | | | | | | | | | | | | | | | | | | |
|  | | | | | | | | | | | | | | | | | | | | | | |
| **Now think about the time during the pandemic since March/April 2020.** | | | | | | | | | | | | | | | | | | | | | | |
|  | | | | | | | | | | | | | | | | | | | | | | |
| Please indicate all types of workstations and the respective hours you have spent working there to perform your work between April 2020 and November 2020. | | | | | | | | | | | | | | | | | | | | | | |
|  | | | | | not at all | | | less than 4 hours | | | 4 to 12 hours | | | 12 to 20 hours | | 20 to 28 hours | | | 28 to 36 hours | | more than 36 hours | |
| Single office | | | | | 🞎 | | | 🞎 | | | 🞎 | | | 🞎 | | 🞎 | | | 🞎 | | 🞎 | |
| Multiperson office (2-4 persons) | | | | | 🞎 | | | 🞎 | | | 🞎 | | | 🞎 | | 🞎 | | | 🞎 | | 🞎 | |
| Multiperson office (5-10 persons) | | | | | 🞎 | | | 🞎 | | | 🞎 | | | 🞎 | | 🞎 | | | 🞎 | | 🞎 | |
| Multiperson office (more than 10 persons) | | | | | 🞎 | | | 🞎 | | | 🞎 | | | 🞎 | | 🞎 | | | 🞎 | | 🞎 | |
| Production workplace  (e.g. factory hall, workshop, assembly line) | | | | | 🞎 | | | 🞎 | | | 🞎 | | | 🞎 | | 🞎 | | | 🞎 | | 🞎 | |
| Laboratory workplace | | | | | 🞎 | | | 🞎 | | | 🞎 | | | 🞎 | | 🞎 | | | 🞎 | | 🞎 | |
| Outdoor / open air workplace | | | | | 🞎 | | | 🞎 | | | 🞎 | | | 🞎 | | 🞎 | | | 🞎 | | 🞎 | |
| Logistics | | | | | 🞎 | | | 🞎 | | | 🞎 | | | 🞎 | | 🞎 | | | 🞎 | | 🞎 | |
| Sales force | | | | | 🞎 | | | 🞎 | | | 🞎 | | | 🞎 | | 🞎 | | | 🞎 | | 🞎 | |
|  | | | | | | | | | | | | | | | | | | | | | | |
| How many days per month (on average) did you work from home during the time you participated in the Corona screening test-offer (April - November 2020) | | | | | | | | | | | | | | | | | | | | | | |
|  | 🞎 | None | | | | | | | | | | | | | | | | | | | | |
|  | 🞎 | 1-5 persons | | | | | | | | | | | | | | | | | | | | |
|  | 🞎 | 6-10 persons | | | | | | | | | | | | | | | | | | | | |
|  | 🞎 | 11-20 persons | | | | | | | | | | | | | | | | | | | | |
|  | 🞎 | more than 20 persons | | | | | | | | | | | | | | | | | | | | |
|  |  |  | | | | | | | | | | | | | | | | | | | | |
| On a normal working day between April 2020 and November 2020, how many people did you have professional contact? | | | | | | | | | | | | | | | | | | | | | | |
|  | 🞎 | None | | | | | | | | | | | | | | | | | | | | |
|  | 🞎 | 1-5 persons | | | | | | | | | | | | | | | | | | | | |
|  | 🞎 | 6-10 persons | | | | | | | | | | | | | | | | | | | | |
|  | 🞎 | 11-20 persons | | | | | | | | | | | | | | | | | | | | |
|  | 🞎 | More than 20 persons | | | | | | | | | | | | | | | | | | | | |
|  |  |  | | | | | | | | | | | | | | | | | | | | |
| On a normal workday between April 2020 and November 2020, how many people did you have contact with for more than 15 minutes? | | | | | | | | | | | | | | | | | | | | | | |
|  | 🞎 | None | | | | | | | | | | | | | | | | | | | | |
|  | 🞎 | 1-2 persons | | | | | | | | | | | | | | | | | | | | |
|  | 🞎 | 3-5 persons | | | | | | | | | | | | | | | | | | | | |
|  | 🞎 | 6-10 persons | | | | | | | | | | | | | | | | | | | | |
|  | 🞎 | More than 10 persons | | | | | | | | | | | | | | | | | | | | |
|  |  |  | | | | | | | | | | | | | | | | | | | | |
| On a normal working day between April 2020 and November 2020, if you had contacts that lasted more than 15 minutes, in how many of these contacts could you maintain a distance of at least 1.50 m? | | | | | | | | | | | | | | | | | | | | | | |
|  | 🞎 | none | | | | | | | | | | | | | | | | | | | | |
|  | 🞎 | 1-2 persons | | | | | | | | | | | | | | | | | | | | |
|  | 🞎 | 3-5 persons | | | | | | | | | | | | | | | | | | | | |
|  | 🞎 | 6-10 persons | | | | | | | | | | | | | | | | | | | | |
|  | 🞎 | More than 10 persons | | | | | | | | | | | | | | | | | | | | |
|  | | | | | | | | | | | | | | | | | | | | | | |
|  | | | | | | | | | | | | | | | | | | | | | | |
| **Questions about test frequency** | | | | | | | | |  | | | | | | | | | | | | | |
|  | | |  | | | | | |  | | | | | | | | | | | | | |
| How often did you get tested by PCR for acute SARS-CoV-2 infection (MCA and elsewhere) between April 2020 and November 2020? | | | | | | | | | | | | | | | | | | | | | | |
|  | | |  | | | | | |  | | | | | | | | | | | | | |
|  | | |  | | | | | |  | | | | | | | | | | | | | |
| Would you like to have been tested more often? | | | | | | | | | | | | | | | | | | | | | | |
|  | 🞎 | Yes | | | | | | | | | | | | | | | | | | | | |
|  | 🞎 | No | | | | | | | | | | | | | | | | | | | | |
|  |  |  | | | | | | | | | | | | | | | | | | | | |
| Why didn´t you get tested more often? | | | | | | | | | | | | | | | | | | | | | | |
|  | 🞎 | I was busy during the time the test stations were open | | | | | | | | | | | | | | | | | | | | |
|  | 🞎 | Overlapping with my regular work schedule | | | | | | | | | | | | | | | | | | | | |
|  | 🞎 | I have used other test offers | | | | | | | | | | | | | | | | | | | | |
|  | 🞎 | The way to the mobile test center was too far for me. | | | | | | | | | | | | | | | | | | | | |
|  | 🞎 | Long waiting times | | | | | | | | | | | | | | | | | | | | |
|  | 🞎 | My direct supervisor was skeptical about testing | | | | | | | | | | | | | | | | | | | | |
|  | 🞎 | Testing did not result in any contribution in terms of my work | | | | | | | | | | | | | | | | | | | | |
|  | 🞎 | I generally see no benefit in the tests | | | | | | | | | | | | | | | | | | | | |
|  | 🞎 | Others: | | | | | | | | | | | | | | | | | | | | |
